# Non-destructive determination of floral staging in cereals using X-ray micro computed tomography (µCT)

**DOI:** 10.1186/s13007-017-0162-x

**Published:** 2017-02-28

**Authors:** Saoirse R. Tracy, José Fernández Gómez, Craig J. Sturrock, Zoe A. Wilson, Alison C. Ferguson

**Affiliations:** 1 School of Agriculture and Food Science, University College Dublin, Dublin, Ireland; 2 Plant and Crop Science, School of Biosciences, University of Nottingham, Sutton Bonington Campus, Loughborough, LE12 5RD UK; 3 Agricultural and Environmental Sciences, University of Nottingham, Sutton Bonington Campus, Loughborough, LE12 5RD UK; 4 Centre for Plant Integrative Biology (CPIB), University of Nottingham, Sutton Bonington Campus, Loughborough, LE12 5RD UK

**Keywords:** *Arabidopsis thaliana*, Barley, Floral staging, Flower development, *Hordeum vulgare* L., Image analysis, X-ray computed tomography

## Abstract

**Background:**

Accurate floral staging is required to aid research into pollen and flower development, in particular male development. Pollen development is highly sensitive to stress and is critical for crop yields. Research into male development under environmental change is important to help target increased yields. This is hindered in monocots as the flower develops internally in the pseudostem. Floral staging studies therefore typically rely on destructive analysis, such as removal from the plant, fixation, staining and sectioning. This time-consuming analysis therefore prevents follow up studies and analysis past the point of the floral staging.

**Results:**

This study focuses on using X-ray µCT scanning to allow quick and detailed non-destructive internal 3D phenotypic information to allow accurate staging of *Arabidopsis thaliana* L. and Barley (*Hordeum vulgare* L.) flowers. X-ray µCT has previously relied on fixation methods for above ground tissue, therefore two contrast agents (Lugol’s iodine and Bismuth) were observed in Arabidopsis and Barley *in planta* to circumvent this step. 3D models and 2D slices were generated from the X-ray µCT images providing insightful information normally only available through destructive time-consuming processes such as sectioning and microscopy. Barley growth and development was also monitored over three weeks by X-ray µCT to observe flower development in situ. By measuring spike size in the developing tillers accurate non-destructive staging at the flower and anther stages could be performed; this staging was confirmed using traditional destructive microscopic analysis.

**Conclusion:**

The use of X-ray micro computed tomography (µCT) scanning of living plant tissue offers immense benefits for plant phenotyping, for successive developmental measurements and for accurate developmental timing for scientific measurements. Nevertheless, X-ray µCT remains underused in plant sciences, especially in above-ground organs, despite its unique potential in delivering detailed non-destructive internal 3D phenotypic information. This work represents a novel application of X-ray µCT that could enhance research undertaken in monocot species to enable effective non-destructive staging and developmental analysis for molecular genetic studies and to determine effects of stresses at particular growth stages.

**Electronic supplementary material:**

The online version of this article (doi:10.1186/s13007-017-0162-x) contains supplementary material, which is available to authorized users.

## Background

There is an increasing awareness of the importance of food security, with an estimated requirement of 50% more food by 2030 [1]. Male fertility in plants is highly sensitive to environmental change such as heat stress, thus fluctuations in global temperature may result in dramatic changes in yield and productivity [2]. To understand the effect of heat stress on pollen development and therefore plant productivity, the analysis of morphological, genetic and biochemical information is required to determine the normal pollen developmental processes. This information can then be used for future modelling and plant breeding to identify strategies to overcome environmental changes [3]. To conduct molecular research of flower development and the stage-specific effects of heat stress accurate staging of flower development is required. This is critical so that traits can be analysed correctly for stress treatments, or for precise collection of materials for molecular, genetic and comparative studies. In monocotyledons (grass-like), flower development occurs internally and therefore there have been limited options available to study the very early stages of inflorescence development, since materials cannot be identified easily without dissection. Dissection is time-consuming and leads to the ultimate destruction of the floral tissue. The ability to accurately stage floral development by a non-destructive technique would be beneficial for a wide range of research in plant reproductive development. In the past experimental plants were characterised by age rather than precise growth stage [4]; such staging is not precise and therefore not suitable for detailed molecular analyses.

In Barley (*Hordeum vulgare* L.), florets begin their development within the pseudostem shoot (a false stem composed of blades and sheaths that surround it), meaning that floral developmental stages cannot be observed or checked without destruction of the floral tissue. Recently Gomez and Wilson [5] produced a non-destructive staging guide based on external morphological features for barley (variety Optic) to allow correct stages to be recognised by external vegetative features. As part of this study they observed that specific anther and pollen stages were tightly linked to spike size [5]. This supported previous observations that spike size was linked to pollen development, which were first reported in the 1980s [6, 7]. While the staging suggested by Gomez and Wilson [5] goes a long way towards selecting appropriately staged material for detailed reproductive studies it has limitations, such as a requirement for controlled growth conditions, variation between genotypes and a high level of developmental variation between tillers [8]. This means that only tillers immediately following the main stem could be used for accurate staging. This staging is also variety specific, with further analysis needed to apply this approach to other varieties, or different growth conditions [5]. Using non-destructive methods that enabled accurate staging of floral development across all tillers, varieties, mutants and in all conditions, would therefore offer a unique insight into plant developmental stages.

X-ray micro computed tomography (μCT) has been used extensively in medical sciences since the 1970s to allow quantification and visualisation of internal structures in 3D in a non-invasive and non-destructive way [9, 10]. The use of this technology has been adopted successfully in many other areas of Science and Engineering. For example in archaeology [11], engineering [12], food science [13] and biology [14]. In the last twenty years it has been widely used in the soil and plant sciences to visualise soil properties [15], the influence of soil microbes on soil structure [16, 17] and plant root development and architecture in 4D imaging [18].

There have been comparatively few studies to visualise and quantify above-ground structure of plants; nevertheless there has been an increased interest in this over the last 6 years [19–26]. One of the main problems for this analysis is the low X-ray attenuation of plant tissue which can result in low image contrast and high levels of image noise. To overcome this problem many studies have used fixing and then infiltration/stained material to provide contrast to enable tissue and cellular differentiation [27]. Through fixation, dehydration and iodine staining high levels of resolution (13.8 and 0.8 μm) were achievable with *Arabidopsis thaliana* (*Arabidopsis*) flower and shoots respectively [20]. Staedler et al. [28] did an in-depth study on *Arabidopsis* and *Marcgravia caudata* flowers using a number of different compounds used previously in transmission electron microscopy (TEM) experimentation and a number of different mounting techniques providing high resolution (1.5 μm) for the tissue. This work highlights the importance of effective sample mounting techniques to maximise image quality at high resolution as even a small movement e.g. due to moisture loss from the tissue, can cause blurred images.

All these methods require lengthy preparation, removal from the plant and results in the destruction of the tissue with the dehydration and fixation stages. More recent analysis has moved towards living tissue, as both the contrast and speed of the µCT scanners has increased, allowing external and internal structures to be visualised without fixing and staining. For example, studies of rice panicle development over time [29]. The main limitation of in vivo analysis is that there is a resolution versus sample size trade off, where fine detail scans require lengthy scanning and processing times, whilst larger samples adversely affect spatial resolution and the ability to differentiate tissue/cells.

To take advantage of the staining for individual tissue differentiation as observed by Staedler et al. [28], we adapted their study to look at living tissue and focused on small molecular stains which would have limited/no impact on plant viability. Another important factor to take into account is the ionizing effect of the X-rays themselves, previous studies by [20] showed that consecutive daily in vivo scanning (10–20 min) for a fortnight resulted in temporal growth inhibition in *Arabidopsis* seedlings. Therefore we aimed to keep our scans to a minimum duration to minimise the X-ray dose received by each sample.

The model plant *Arabidopsis* was used to optimise the X-ray µCT scanning technique and to enhance image quality different contrast agents were explored. This knowledge was then transferred to the crop Barley to optimise monocot imaging. To date there have been no previous studies of aerial parts of Barley using X-ray µCT scanning, this is therefore the first report of analysis of dissected Barley spikes and aerial parts from pot grown Barley plants. This novel method benefits from utilising the non-invasive technique so that we can obtain a 3D visualisation of floral development in the same flower over a growth period, and allows for non-destructive, quick and accurate staging of material.

## Methods

### Plant materials

The external whole inflorescence and single floral buds of *Arabidopsis thaliana* [wildtype (*wt*) ecotype Col-0] were used as it is a widely studied model plant; it comprises compact, complex organs and has been used previously in X-ray µCT studies [28]. *Arabidopsis* was grown in Levington M3 (The Scotts Company Ltd, UK) compost supplemented with Met52 (Novozymes, France) and nematodes (Sygenta Bioline, UK) under controlled conditions of 22/2 h photoperiod at 21/17 °C light/dark cycle. Flower development was based on the flower stages by Smyth et al. [30] and further expanded by Sanders et al. [31]. Whole plant and internal spikes of Barley (*Horedum vulgare* cv. Optic) were scanned to assess the penetration of the X-rays into thicker and denser tissue barriers. Spring barley, Optic, was sown 3 plants to a pot in John Innes number 3 (John Innes, UK) compost and then transferred to Levington C2 compost after 3 weeks; they were grown under controlled conditions of 16/8 h photoperiod 15/12 °C light/dark cycle (80% relative humidity). Barley was observed after the onset of elongation stages to heading to allow the reproductive development to be followed. Flower development was based on flower stages as described by [5]. Spike size was measured from the base of the most basal floret to the tip of the most apical floret.

### Infiltration

To enhance visualisation of plant tissue contrast agents were used. Iodine (Lugol’s solution) and bismuth acetate were selected based on existing studies as least toxic to a developing plant and small enough to be taken up by the root system. Plants were treated with the contrast agents by watering two days prior to scanning (*Arabidopsis*) or one week prior to scanning (Barley). Treatments included 1% Lugol’s solution, 5 mM bismuth solution, and water (non-treated control).

### Mounting

**Fig. 1:**
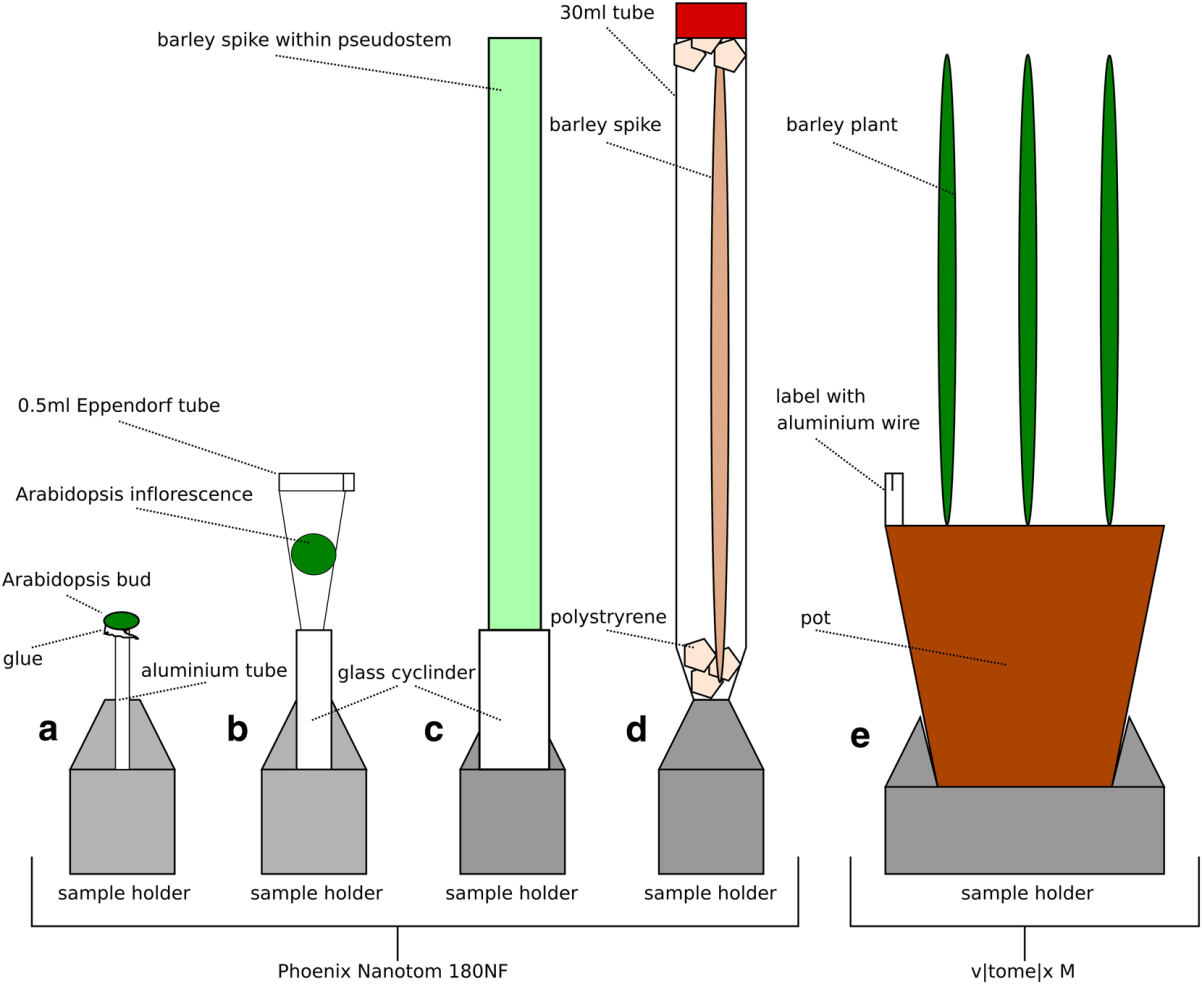
Sample mounting techniques for the different samples.* a* Mounting of single *Arabidopsis* buds onto aluminium tube using glue.* b* Whole *Arabidopsis* inflorescence was mounted into a 0.5 ml Eppendorf tube, making sure the inflorescence was supported firmly and unable to move. *c* The pseudostem was cut above and below the developing barley spike and placed directly into a glass cyclinder to hold it secure in the scanner. *d* The barley spike was removed from the pseudostem and to keep it secure during the scan it was placed in a 30 ml tube with polystryrene at the* base* and* top* of the tube to hold it in position. *e* Growing barley plants were placed directly into the scanner. *Each pot* was labelled with a plant label with an aluminium strip(s) of wire so that *each pot* could be indentified from the scanned image

Each sample was mounted differently within the two scanners to ensure that materials were fixed with minimal movement occurring during the scan (see Fig. 1). *Arabidopsis* flowers were separated into single buds and scanned individually. Buds with a diameter 1–10 mm were mounted on an aluminium tube with glue and were either scanned immediately or left for 20 min to equilibrate.

After infiltration treatments of 2 days, *Arabidopsis* whole inflorescence were manually removed from the plant and placed into 0.5 ml Eppendorf tubes to secure the sample and prevent movement throughout the scanning. The tubes were then mounted onto a glass cylinder and left for 20 min to equilibrate before scanning.

After infiltration treatments of 1 week, Barley spikes were dissected from the main tiller and scanned individually. These were either kept within the pseudostem (cut above and below the spike) to provide structure and prevent movement within the scan time, or the spike was manually removed from the pseudostem. These were then either mounted directly into the machine or placed within a 30 ml tube respectively; polystyrene was used to secure the spike in a vertical position within the tube and prevent movement, the tube was then mounted directly into the machine for the scan.

### Scanning

X-ray µCT scanning of the *Arabidopsis* and Barley spikes was performed using a Phoenix Nanotom 180NF (GE Sensing & Inspection Technologies GmbH, Wunstorf, Germany). The scanner consisted of a 180 kV nanofocus X-ray tube fitted with a tungsten transmission target (diamond exit window) and a 5-megapixel (2316 × 2316 pixels) flat panel detector (Hamamatsu Photonics KK, Shizuoka, Japan). Scans of the whole Barley plant were performed using the v|tome|x M 240 kV X-ray µCT scanner (GE Sensing & Inspection Technologies GmbH, Wunstorf, Germany). Scanning conditions are detailed in Table 1, *Arabidopsis* scans took 29 min, while barley spikes took 32 min, and Barley whole plant took 10 s to obtain a quick snapshot image from the radiograph. The whole Barley plant was moved within the scanner to provide close up images of each spike being monitored so that accurate measurements could occur.

**Table 1:** Material, treatment, scanner and scanning parameters for each scan performed

Material	Treatment	Scanner	kV	μA	μm	P	ms	T	Bin	Av/Sk	Type
*Arabidopsis* bud	N/A	Nanotom	45	180	2.25	1200	250	15	2 × 2	3 × 0	Fast
*Arabidopsis* inflorescence	1% Lugols solution/5 mM bismuth/water	Nanotom	45	180	2.25	1200	250	15	2 × 2	3 × 0	Fast
Barley spike	5 mM bismuth/water	Nanotom	100	100	3.65	1200	500	24	1 × 1	1/0	Fast
Barley whole plant	N/A	V|tome|x M	60	250	30	2398	200	24	1 × 1	1/0	Fast/multi

kV = X-ray source energy (kV); µA = X-ray source current (µA); P = Number of projection images; ms = detector timing (ms); T = total scan time (mins); Bin = Detector pixel binning; Av/Sk = Number of projection image averaging and skip; Type = Scans were made in FAST mode where the sample is subject to continuous rotation during projection image acquisition

### Computational analysis

**Fig. 2:**
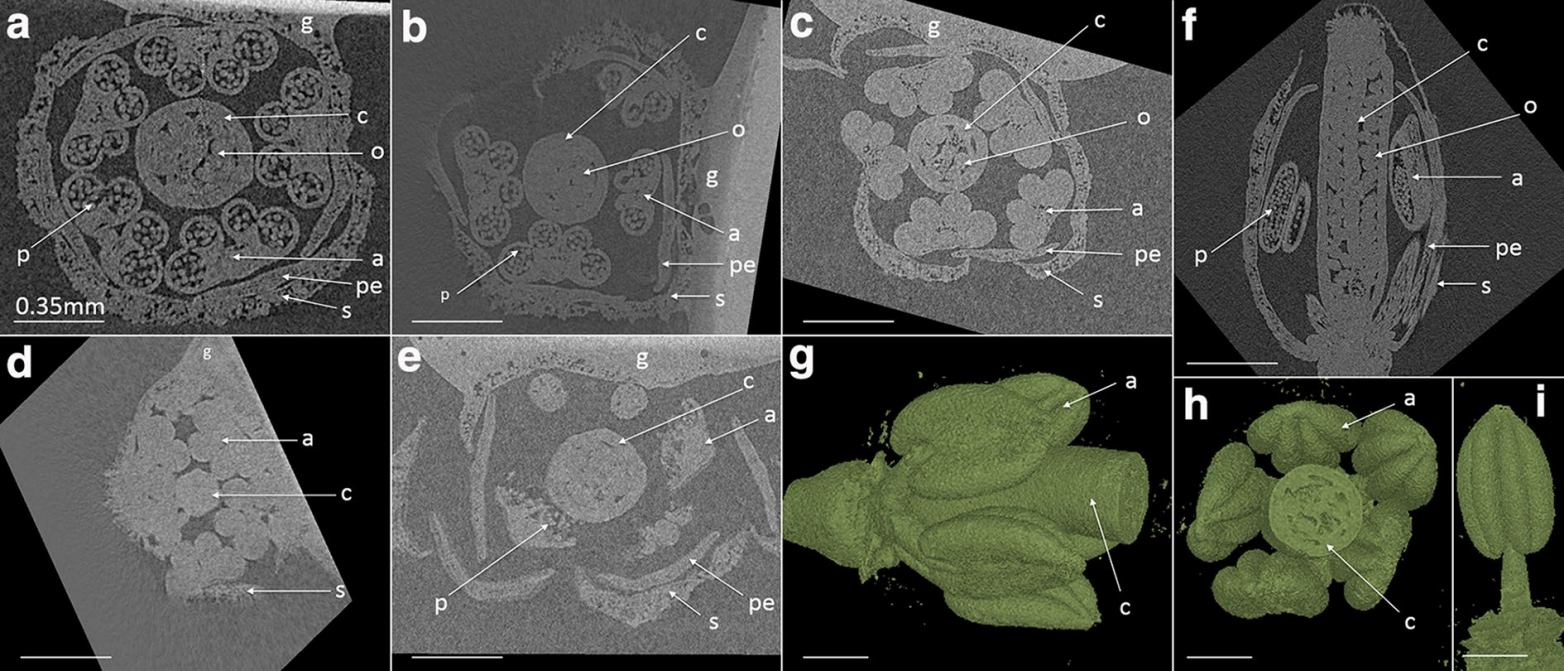
X-ray µCT XY cross section image of *Arabidopsis* single buds at different developmental stages. Flower staging based on [30]; Flower stages 11 (**a**, **f**), 10 (**b**), 9 (**c**, **d**), 13 (**e**) showing individual organs, pollen and ovules. 3D representative images (**g**, **h**) of bud, and of an isolated anther at flower stage 11 (**i**). Within the images; carpel (*c*), anther (*a*), petal (*pe*), sepal (*s*), ovule (*o*), pollen (*p*), glue to attach bud to glass needle (*g*). *Scale bar* is 0.35 mm

**Fig. 3:**
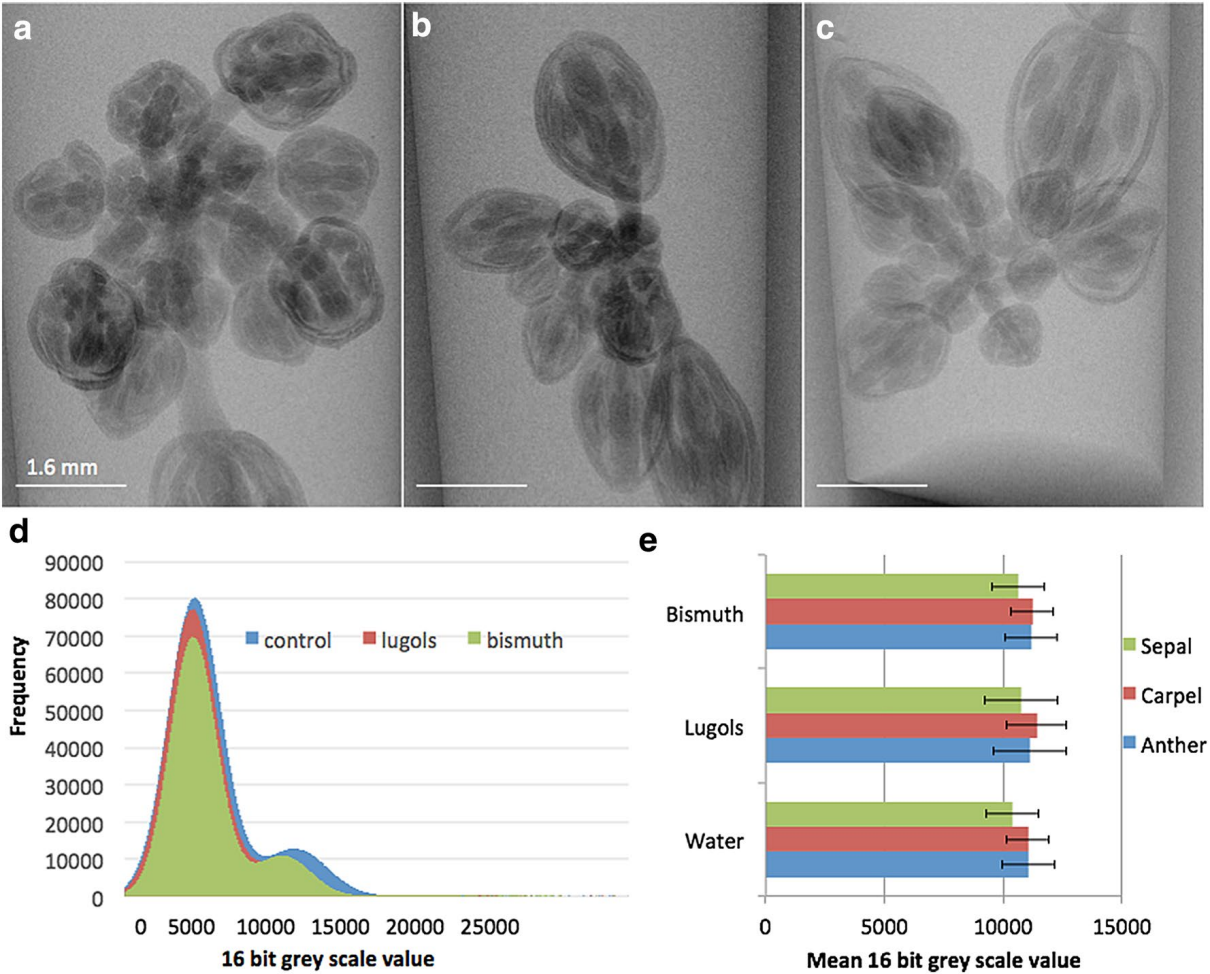
X-ray µCT radiograph image of whole *Arabidopsis* inflorescence using different contrast agents and their density gradients. Different contrast agents were used; water control (**a**), Lugols (**b**), and Bismuth (**c**); *scale bar* is 1.6 mm. Frequency histogram of the image greyscale values using different contrasting agents in *Arabidopsis thaliana* floral buds scanned using the Phoenix(R) Nanotom(R) X-ray μCT scanner in the whole image (**d**), and mean values for tissue specific (Anther, Carpel and Sepal) (**e**). *Error bars* show standard deviation

**Fig. 4:**
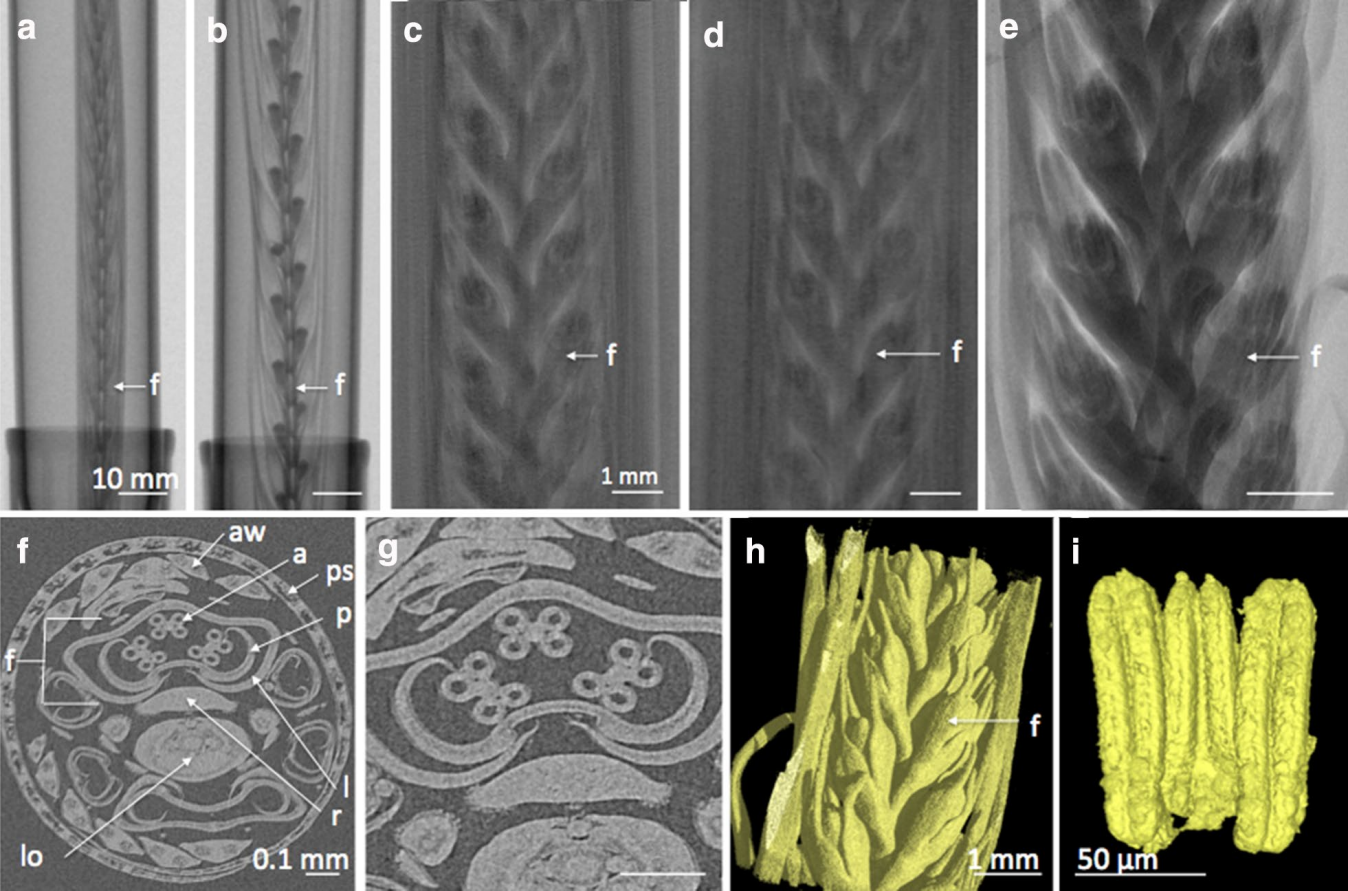
X-ray µCT radiographs, XY cross sections and 3D rendered images of Barley spikes manually removed from the tiller. **a** Barley spike at 50 mm, **b** mature Barley spike at 70 mm, **c** close up scan of control (water) spike, **d** close up scan of Bismuth treated spike, **e** close up µCT scanned of Barley spike unsheathed, **f** XY cross section of the control (water) spike still sheathed, **g** close up of cross section highlighted three anther structures, **h** 3D rendered image of unsheathed floral bud using X-ray µCT, **i** Dissected 3D image of Barley anther. Within the images; floret (*f*), anther (*a*), palea (*p*), rachella (*r*), lemma (*l*), lodicule (*lo*), awn (*aw*), pseudostem (*ps*). *Scale bar* **a**, **b** is 10 mm; **c**–**e**, **h** is 1 mm; **f**, **g** is 0.1 mm; **i** is 50 µm

The µCT image slices were reconstructed to provide a 3D visualisation of floral structure for *Arabidopsis thaliana* and Barley spikes. The resultant images were analysed to quantify the level of obtainable detail at the organ, tissue and cellular level. The reconstructed µCT volumes were visualised and quantified using VG StudioMAX© 2.2 (Volume Graphics GmbH, Heidelberg, Germany), after a graphical filtering of median (x = 3 pixels). This allowed segmentation of the floral organs and also separately of the anther (Figs. 2, 3, 4). Samples were normalised to two reference materials (air and material) for Arabidopsis, and just one reference (air) for Barley, and using the VG StudioMAX© software greyscale values of the whole bud/spike, as well as different organs in the different treatments were analysed to determine the effectiveness of the contrasting agent (Fig. 3). ImageJ 1.48v (ImageJ, U. S. National Institutes of Health, Bethesda, Maryland, USA) was used to measure the spike sizes in the different tillers of the non-treated whole barley plants, based on a scale marker attached to each analysed tiller, allowing pixel to size quantification.

### Histological analysis of anther development

Florets from the middle zone of the spike were collected from different-sized spikes and fixed in paraformalehyde and then embedded in technovit resin in order to determine reproductive development stage. Fixation and staging occurred following the protocol of [29], sections were obtained using the Bright 5040 microtome (Bright Instruments, UK). Slides were stained with 0.05% (w/v) toludine blue prior to imaging.

## Results

### In-depth analysis of *Arabidopsis* single buds

Different floral development staged buds (based on flower staging by [30, 31], see Table 2) were analysed to evaluate the application of μCT technique on living tissue without fixation. Using the scanning conditions (Table 1) for *Arabidopsis* within the Nanotom, samples were easily detected at the organ level, and to some respect the tissue level for pollen and ovules giving a potential level of resolution of 2.25 μm, and actual spatial resolution of 10 µm (Fig. 2; Additional file 2: Video S1). Air space was also detectable within the leaf and petals. However scanning resolution and image quality were highest at flower stage 10–12 (Fig. 2a, b, f), with the dehiscence buds (flower stage 13) (Fig. 2e) and younger buds (flower stage 9) (Fig. 2d) experiencing movement during the scan, thus producing blurred images. For the youngest buds it was difficult to detect the different tissues and organs due to the fact that the tissue was tightly packed together. Despite movement within the oldest tissue causing blurred images, the different organs were easily identifiable and released pollen could be detected (Fig. 2e; Additional file 3: Video S2). This shows that while the µCT scanning technique is able to produce sliced images of *Arabidopsis* without the requirement for fixing and sectioning, it is unable to provide detailed information to allow for specific Anther or Pollen stages [31], although flower stages were identifiable [30].

**Table 2:** Arabidopsis and barley flower stage reference guide, showing flower stage in relation to anther/pollen development

Arabidopsis flower stage	Anther/pollen development	Barley flower stage	Anther/pollen development
8	Sporogenous cells and three anther cell layers	Spike 0–1 cm Zadoks stage 31	Sporogenous cells and three anther cell layers
9	Four anther cell layers, pollen mother cell formed and undergo meiosis	Spike 1–2 cm Zadoks stage 32	Four anther cell layers, pollen mother cells formed
10	Pollen mother cell meiosis completed, and single microspores released from the tetrad	Spike 2–3 cm Zadoks stage 33	Pollen mother cell meiosis
		Spike 3-4 cm Zadoks stage 33.5	Released single microspores from tetrad
11	Single microspores and tapetum undergoes degeneration	Spikes 4–5 cm Zadoks stage 34	Single microspores and middle layer undergoes crushing
		Spikes 5–6 cm Zadoks stage 35–37	Vacuolated microspores. Tapetum degenerating
12	Pollen mitosis I, and tapetum degeneration almost completed	LFE1 Spikes 6–8 cm	Tapetum degeneration finished. Pollen mitosis I begins
		LFE2 Spikes ~9–10 cm	Pollen mitosis I completed
13	Binuclear pollen. Pollen mitosis II occurs, forming tri-nucleated pollen	LFE3 Spikes ~9–10 cm	Binuclear pollen. Pollen mitosis II occurs.
		LFE4 Spikes ~9–10 cm	Tri-nucleated pollen and anthesis taking place

Table based on staging by [5, 30–32]

Flower stage 11 (Fig. 2a) were further analysed as it gave the highest resolution images, and a 3D model was created in VG StudioMAX© using image analysis techniques (segmentation using the Region Grower tool VG StudioMAX©), petals and sepals were easily virtually removed to give the internal flower structure. This shows the amount of detail at the organ level achievable by µCT scanning. Differences in organ sizes between different treatments or mutants could be measured in this way rather than by dissection.

### Analysis of *Arabidopsis* inflorescence

Whole inflorescence of *Arabidopsis* was used to test the detection capabilities of the μCT scanner on living tissue using different contrast agents (Fig. 3a). Under the employed scanning conditions, samples and separate organs were detectable using all treatments. Greyscale intensity was used to observe the effect of different contrast agents; the histogram showed that both treatments had little effect on the overall contrast (Fig. 3b). These images were then further analysed to see if the contrast agents could generate differences between different organ structures in living tissue by using transverse sections of the *Arabidopsis* buds. Examining the average mean values across at least five anthers, carpels and sepals for each treatment showed that there were no statistical differences in the contrast between the different organs. Therefore there were no advantages of using a contrast agent compared to the water control for analysis of flower development in living tissue in our study.

### Analysis of Barley spike

Barley spikes were manually removed and placed into a 30 ml tube to hold the sample static for imaging by the Nanotom (Fig. 1c, d). Figure 4c, d, f, g were kept sheathed with the Barley plant cut above and below the spike, while Fig. 3a, b, e were unsheathed before scanning leaving only the barley spike. Using the scanning conditions (Table 1) for Barley within the Nanotom, samples were easily detected at the organ level. No segregation at the tissue level could be achieved, with a potential resolution of 3.65 μm and actual resolution of 15 µm was achieved (Fig. 4). However, due to the denser tissue and thicker stem there was less movement during the thirty minute scan which resulted in sharper images. The µCT scanning technique was able to produce sliced images of Barley without the requirement for fixing and sectioning, but it was unable to provide detailed information to allow for specific anther or pollen stages and earlier flower stages (Additional file 4: Video S3). Similar to the *Arabidopsis*, no contrast improvements between the sheath, anther and carpel tissue were found (Additional file 1: Figure S1). However the Bismuth contrast agent gave a wider and shifted histogram pattern showing that it was taken up into the tissue, with an obvious peak for air (first peak) which was not detectable in the water control (Additional file 1: Figure S1).

### Analysis of Barley plant for flowering stage floral inflorescence

**Fig. 5:**
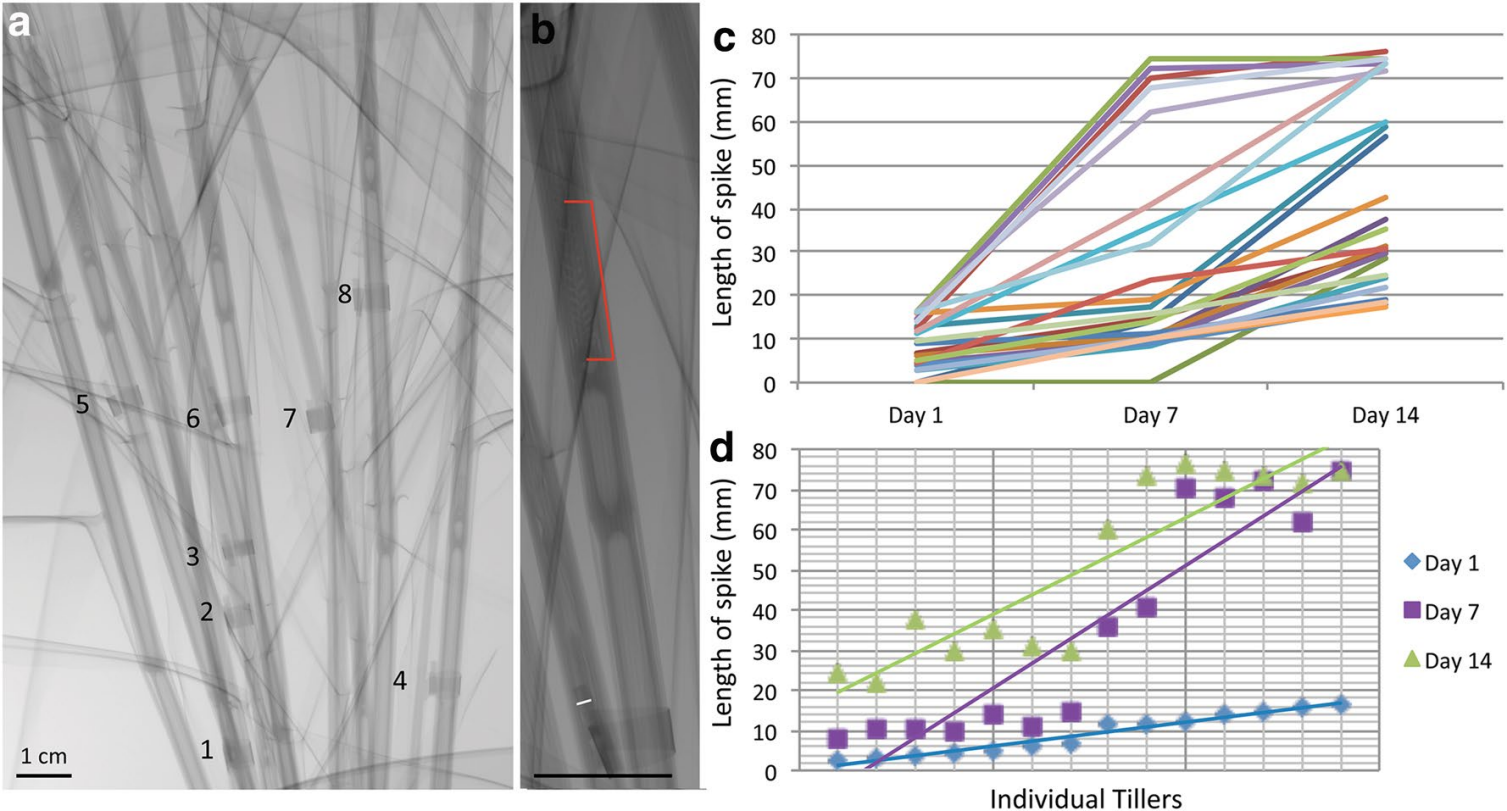
*In planta* radiograph image of Barley and spike size analysis. **a** Whole plant image slice of Barley, showing 8 tagged tillers. **b** Close up image of the whole Barley showing floral spike size highlighted by *red segment* and *white line* represents fixed marker (1.2 mm). *Scale bar* indicates 1 cm. **c** *Graph* showing all measured spike sizes by CT scanning and their growth over 2 weeks, measured at Day 1, 7 and 14. **d** *Graph* showing measured spike sizes for individual secondary tillers arranged by their original size at Day 1, observing growth patterns over two weeks, measured at Day 1, 7 and 14

**Fig. 6:**
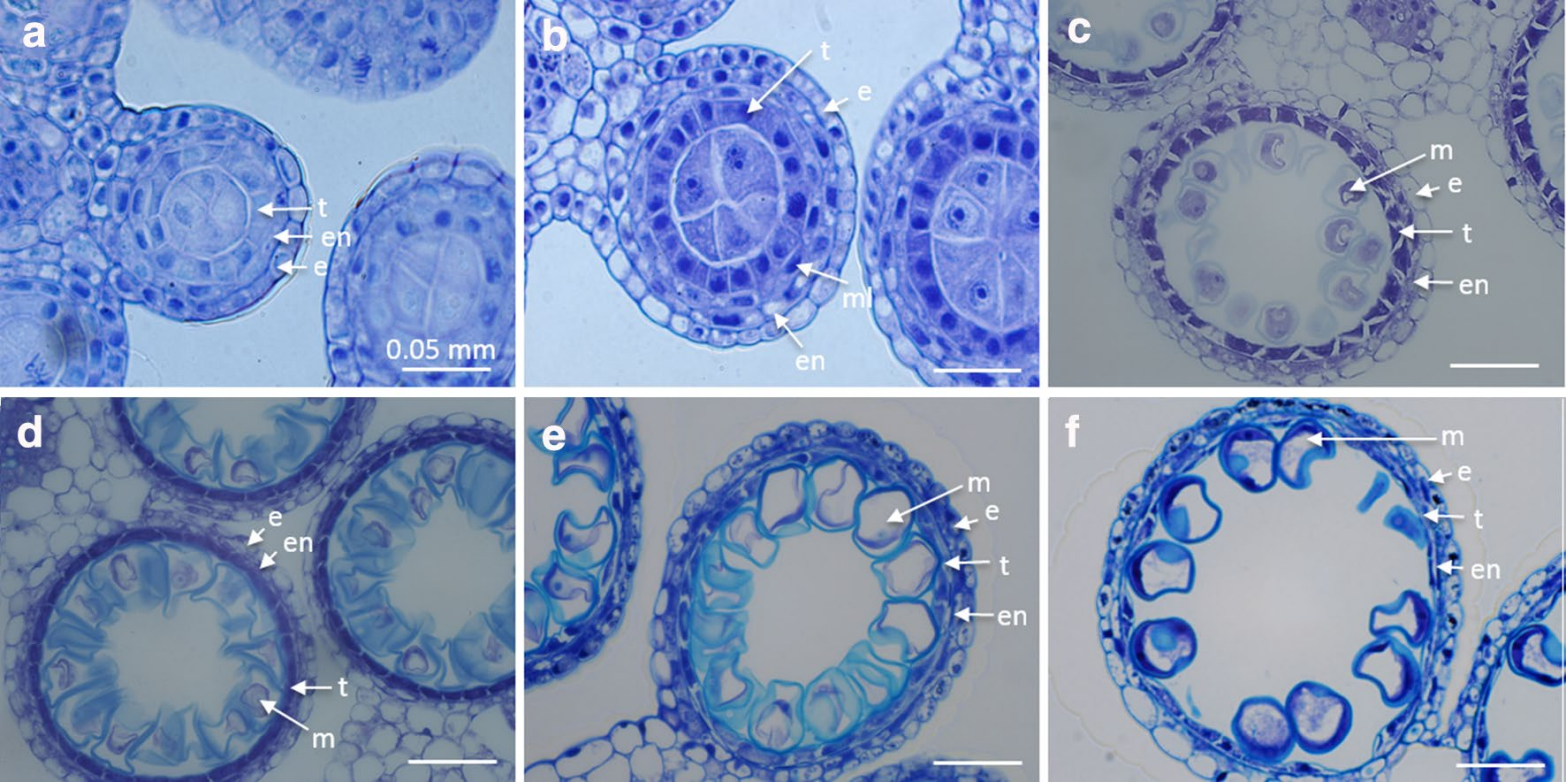
Barley anthers fixed, sectioned and stained with toluidine *blue* to determine flower stage. Florets extracted from the* middle* of the spike at; **a** 11 mm spike showing three cell layers and primary sporogenous cells at Zadoks stage 31; **b** 17 mm spike showing four cell layers and secondary sporogenous cells to pollen mother cells at Zadoks stage 32; **c** 35 mm spike showing released microspores and tapetum becomes vacuolated at Zadoks stage 34; **d** 53 mm spike showing microspores becoming vacuolated and middle layer breakdown at Zadoks stage 36; **e** >60 mm spike before LFE1 showing tapetal breakdown; **f** >70 mm spike at LFE1-2 showing tapetum breakdown and microspores going through mitosis I. Within the images; tapetum (*t*), endodermis (*en*), epidermis (*e*), middle layer (*ml*), microspores (*m*). *Scale bar* is 0.05 mm

The v|tome|x M 240 kV µCT system was used to scan the whole Barley plant to identify floral stage (based on staging by Smyth et al. [30], see Table 2) *in planta* without dissection (Table 1; Fig. 1e). To limit the X-ray dose to the plant, a single radiograph of the Barley plant was acquired consisting of 32 integrated images. This process took 10 s per radiograph with the spike being easily identifiable using this method (Fig. 5). To allow tillers to be monitored over time and accurately measured, a fixed marker was attached to each tiller, and an overview wider scan view was taken of each plant at the start of scanning so individual tillers and spikes could be monitored over the progressive weeks (Fig. 5a). For accurate measurement of the spike, further close up images of each tiller were taken, with the pot being rotated to allow the best view of each tiller with no other tillers obscuring it if possible (Fig. 5b). This increased the total time to take the necessary pictures of each Barley plant to approximately 5 min. Floral development of each individual spike was observed weekly over a 14 day growth period to give three time points (1, 7 and 14 days). Twenty five individual tillers were examined for floral development, over three separate Barley plants looking at all the tillers going through flower development within the plants. Different sized spikes showed a general trend in growth over the two week period, however there were outliers, with some developing quicker or slower than the rest (Fig. 5c, d). Cytological analysis was used to determine staging based on anther and pollen development stage within the dissected floret (Fig. 6) [5]. This combined analysis of µCT and cytological features indicates that the majority of plants were at 0–10 or 10–20 mm at day 1 showing sporogenesis tissue with three cell layers (Fig. 6a) and four cell layers (Fig. 6b), Zadoks stages 31 and 32 respectively. These spikes progressed to pollen mother cell meiosis (20–30 mm) (Zadoks stage 33) and microspore release (30–40 mm) (Zadoks stage 34) (Fig. 6c) for the spike at three cell layers and mitosis I and II for the spikes at four cell layers (>70 mm, last flag elongation (LFE) 1–2 and LFE3; Fig. 6f) during the two week observation [5, 32]. While there was a general trend that larger starting size of the spike at day 1 lead to a certain size spike at day 7 and day 14 (Fig. 5c), this was not true for all spikes, with a few going through development quicker than average and others slower. This appears to be due to the tiller location, since if we removed the oldest tiller (main tiller) and the youngest tillers from the analysis the trend was more striking (Fig. 5d). It also highlights a developmental difference between those spikes at four cell layers compared to three cell layers, with the older spikes able to complete pollen development quicker (within 7 days). Whereas all the spikes at the three cell layers, were at four cell layers by 7 days but progressed slower in their development only getting to microspore release stage, rather than complete pollen development, within the next 7 days. In the field Barley produces a lot less tillers, and this slower development could highlight the energy expenditure to allow this number of tillers to go through development and set seed [33]. Despite differences in timing of flower development, the early floral stage could be predicted simply by spike size (Figs. 5, 6). The use of µCT scanning could allow flower stage of all the tillers within barley to be identified and therefore the specific stage of each tiller during a particular treatment (heat stress for example) could be determined, which would allow end point analysis to occur for each of these developmental stages separately enabling an accurate evaluation of the results. This does however highlight a problem at the later stages of flower development (mitosis I onwards) where the spike size does not change remarkably, however these stages LFE1–3 are easily distinguishable by external features of the last flag sheath, and spike, as the spike has emerged from the pseudostem [5].

## Discussion

Knowledge was gained regarding the visualisation of Barley and *Arabidopsis* aerial organs using X-ray µCT scanning technique. The model plant *Arabidopsis thaliana* was used to optimise scan settings, mounting techniques and contrast agents for internal organ identification and staging of flower development. Mounting techniques that held the samples secure during the scan to minimise movement presented the clearest images (Eppendorf tube and 50 ml tube for Arabidopsis and Barley respectively). These techniques seemed to have little impact on the resolution when compared to those that were mounted directly. We have shown that there can be problems caused by movement within the scan both by the mechanical movement whilst being scanned, but also due to dehydration and water loss during the scan. We have found that allowing the tissues to acclimatise once they have been removed from the plant for at least twenty minutes ameliorated this effect for the older bud tissue in *Arabidopsis*. Movement during the scan was less of an issue for monocotyledon plants such as Barley, since the tiller is sturdy and limits movement and dehydration of the spike.

Contrast agents considered were used by [28] however as their study destroyed the tissue by fixation and staining, it was important to select those compounds which had small molecular size so that they could be taken up by the root system and distributed within the plant. They also had to have a low level of known toxicity to prevent deleterious side effects. Lugol’s solution and Bismuth were chosen based on these criteria and Bismuth was shown in previous studies to give differential contrast to the filament, petals and to a lesser extent sepals and ovules [28]. Both Iodine and Bismuth are used for µCT scanning for animal/human studies due to the contrast and low toxicity [34]. In our study we have demonstrated that in Arabidopsis, both Bismuth and Lugol’s solution gave a similar profile to the water control and it is possible that these agents were not taken up successfully by the plant over the 2 days infiltration. Unsurprisingly for both of these treatments there was also no differentiation between different tissue layers. In Barley Bismuth was taken up successfully by the plants and provided an increase of X-ray attenuation of the tissue, although it did not lead to differentiation between tissues. Therefore under the conditions used the contrast agents did not enhance the differences between the living tissue flower organs which would allow for their automatic segmentation as observed by [28]. The whole Barley plant growing in pots were therefore observed with no addition of contrast agents to determine floral staging by spike size.

To validate the imaging technique, florets were collected, sectioned and DAPI stained to enable detailed staging through cytological analysis. This allowed the determination of floral stage for the corresponding spike sizes, showing that a particular spike size is linked to flower development stages. Previous work by Gomez & Wilson [5] developed a modified Zadoks scale for barley reproductive development that was linked to vegetative features at different stages of flowering development, this showed that spike size is correlated to particular stages in development. The work presented here builds on this and allows accurate staging simply based on spike size measured by a quick 1 min scan and a simple measurement of the spike length by computational analysis. With 0–10 mm showing sporogenesis tissue with three cell layers, 10–20 mm showing four cell layers, 20–30 mm showing pollen mother cell meiosis, 30–40 mm showing microspore release and mitosis I and II in the 70 mm spikes (Fig. 6). The latter stages showing similar spike sizes and therefore external physiological features need to be used to differentiate between these stages, these are easily identifiable both visually and in the µCT scan as the spike has matured out of the pseudostem as described by Gomez and Wilson [5].

In this article we demonstrate the feasibility of using X-ray µCT for non-destructively visualising and quantifying the internal organ (spike) size of Barley to allow for accurate staging of floral tissue. The rapid 1 min scans require minimal preparation and computational analysis to enable accurate development staging. This has a major advantage over current staging methods that are destructive resulting in the death of the tiller for spike measurement and cytological analysis. Cytological analysis is also time consuming and requires a number of different steps, such as fixation, sectioning and staining before eventual microscope analysis of the stages. This means that accurate staging often occurs post-sample collection. Both of these current methods provide no opportunity for on-going developmental analysis, or subsequent sample collection if staging has been premature. The added benefit of this method is that the stage of all the spikes within the barley tillers can be established and therefore multiple stages can be observed in the same plant under particular treatments/mutants/cultivars. This is more consistent with what would happen in the field with environmental stresses affecting tillers differently depending on the current developmental stage of the flower.

This approach enables rapid and accurate staging of materials for collection for molecular analysis; this is critical given the specificity of gene expression over these developmental transitions and the requirement for accurate collection of materials, this is particularly the case when materials are limited, for example during transgenic line analysis. This technique would also be useful when looking at the effects of abiotic and biotic stress during plant flower development, as individual tillers could be monitored separately to see what effect the stress has over whole plant yield and yield during a particular flower stage. Temperature variability and extreme fluctuations (heat stress) have been shown to affect plant yield [35]. Studies in high temperature (as well as other stresses) are essential to help identify germplasm that is more tolerant of abiotic stress and also the adaption of agricultural practices to mitigate the impact of such stresses on global food security, such as shifting sowing time, and changing cultivars to reflect the changing environmental conditions.

Furthermore, this approach could be developed into phenotyping platforms which combined with data such as plant height, chlorophyll content, root structure etc. would allow high-through put large-scale data to be correlated with fertility traits to give an overview of crop development and yield. Automated plant phenotyping is rapidly becoming mainstream, with buildings being designed specifically for automated phenotyping to measure a number of different aspects of plant growth, using imaging and different tools to record these [36]. Phenotyping approaches that enable the collection of this information alongside other data on plant physiology and development offer a more holistic analysis of growth, thus enabling links between general plant growth, reproductive development and final yield to be made. This technique for staging flowers is also directly applicable to other monocot species where the flower develops internally, such as wheat and rice, to aid scientific research.

We have shown that while there is regulation of floral development between spike size and flower development, the progression of individual tillers is variable. This is likely to be based on the environment and other features such as tiller position, for example primary versus secondary, total number of tillers and where they are in development can effect flower development [33, 37]. This differential flower development and spike growth has been previously observed and is the reason why scientific observations are frequently limited to primary and secondary tillers for accurate development progression when observing traits. The use of the µCT scanning technique means that the particular stage of all tillers can be observed without relying on them developing at a certain rate under controlled conditions, giving an in-depth analysis of floral stage and final yield.

X-ray μCT can be used for visualisation of both *Arabidopsis* and Barley without any prior fixation or staining allowing living plants to be analysed progressively as they develop. As X-ray μCT scanners improve it will allow for rapid screening of plant tissue development in 3D over time. In X-ray μCT studies there will always be a trade-off between magnification and measured volume, however an advantage of using this technique for plant aerial parts is that because the parts are individually smaller they can be placed close to the X-ray tube to enable high resolution, but still be grown under field conditions or in field representative pots. This has been an issue for using μCT analysis for root and soil studies, as the whole diameter of the pot is usually included in the scan to extract the full root system architecture, so limits the achievable resolution.

## Conclusion

X-ray μCT can be used for living plants to be analysed progressively as they develop, for 2D (sliced sections), 3D (structure) and 4D (over time) analysis. This study has shown that prior fixation or staining is not required for a model dicot and monocot plant species (Arabidopsis and Barley). X-ray μCT can be used to accurately stage flower development non-destructively which is especially important in the monocotyledon species where the flowers begin their development internally. Accurate staging based simply on spike size was measured by a quick 1 min scan. The rapid 1 min scans require minimal preparation and computational analysis to enable accurate development staging. This approach could be incorporated into high-throughput plant phenotyping platforms. Combination of this technique and method has the opportunity to support research into flower development and further understanding of plant developmental responses to biotic and abiotic stresses.

## Additional files

**Additional file 1: Figure S1.** Histogram of greyscale value of Barley spikes. Histogram of greyscale value of density gradients using different contrasting agents in Barley spikes scanned using the Phoenix^®^Nanotom^®^X-ray μCT-scanner in the whole image (A), and mean values for tissue specific (Anther, Stamen and Sheath) (B). Error bars show standard deviation.

**Additional file 2: Video S1.** Sliced stack XY images of *Arabidopsis* bud flower stage 11.

**Additional file 3: Video S2.** Sliced stack XY images of Barley spike sheathed.

**Additional file 4: Video S3.** Sliced stack XY images of Barley spike sheathed.

## Abbreviations

(3D): 3-dimensional
(4D): 4-dimensional
LFE: last flag elongation
TEM: transmission electron microscopy
µCT: micro computed tomography
