# Supplementary figures and images for: Non-destructive determination of floral staging in cereals using X-ray micro computed tomography (µCT)
Source: Plant Methods. 2017 Feb 28;13:9. doi: 10.1186/s13007-017-0162-x (PMC5331626; doi:10.1186/s13007-017-0162-x)

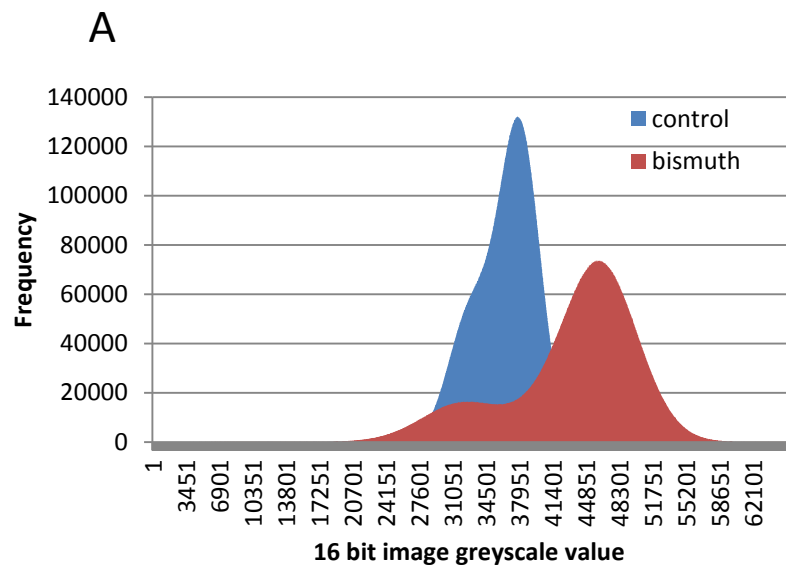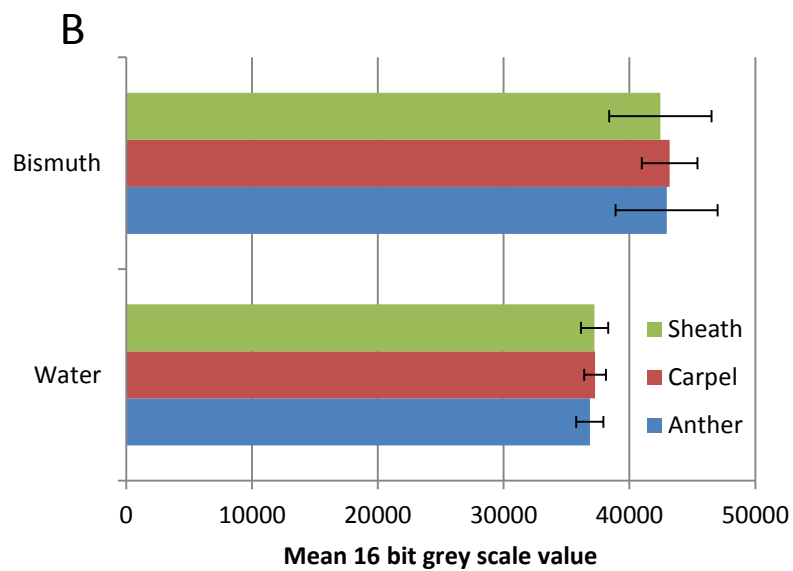

Supplement: Supplementary file 1 — Additional file 1: Figure S1. Histogram of greyscale value of Barley spikes. Histogram of greyscale value of density gradients using different contrasting agents in Barley spikes scanned using the Phoenix®Nanotom®X-ray μCT-scanner in the whole image (A), and mean values for tissue specific (Anther, Stamen and Sheath) (B). Error bars show standard deviation. [file 13007_2017_162_MOESM1_ESM.pdf]
